# Balanced Ionic Conductivity and Permselectivity of Cation Exchange Membranes Prepared from Sulfonated Poly(ether sulfone)

**DOI:** 10.1021/acsomega.5c09018

**Published:** 2025-12-19

**Authors:** Hussien K. Srour, Mizuki Inoue, Edhuan Ismail, Minato Higa, Mitsuru Higa, László Szabó, Izumi Ichinose

**Affiliations:** † Research Center for Macromolecules and Biomaterials, 52747 National Institute for Materials Science, 1-1 Namiki, Tsukuba 305-0044, Japan; ‡ Graduate School of Science and Technology for Innovation, 13150 Yamaguchi University, 2-16-1 Tokiwadai, Ube, Yamaguchi 755-8611, Japan; § Center for Advanced Materials, Forestry and Forest Products Research Institute, 1 Matsunosato, Tsukuba, Ibaraki 305-8687, Japan

## Abstract

Sulfonated poly­(ether sulfone) (S-PES) was synthesized through the postsulfonation of poly­(ether sulfone) (PES) with chlorosulfonic acid as the sulfonating agent. The degree of sulfonation (DS) of the produced S-PES was controlled between 13.2 and 36.2% by adjusting the reaction time. The DS values were determined by elemental analysis, ^1^H NMR, and titration against 0.01 M NaOH. The presence of sulfonic acid groups was confirmed by using ^1^H NMR and FT-IR analyses. The produced S-PES with different DS values was cast into membranes. The performance of these materials as cation exchange membranes (CEMs) was evaluated in detail, in light of their ion exchange capacity, water uptake, hydration number, charge density, contact angle, thermal and long-term stability, ionic conductivity, and permselectivity. The properties of the obtained membranes were compared to various reported CEMs, including industrial benchmarks such as Nafion-117, Fuji CEM, FKS-20, CMX, and CSE. We demonstrate that our work achieved the best balance between ionic conductivity and permselectivity through finely controlled DS optimization. The best-performing membrane was found to have a DS of 32.4% with a significantly high ionic conductivity of 16.85 mS cm^–1^ and a high permselectivity of 98.0% in 0.5 M NaCl. Our work sheds light on the crucial interplay between various membrane properties as the degree of functionalization varies stepwise. The fabricated membranes are promising candidates for advancing electrodialysis-based desalination and salinity-driven renewable energy production technologies.

## Graphical abstract

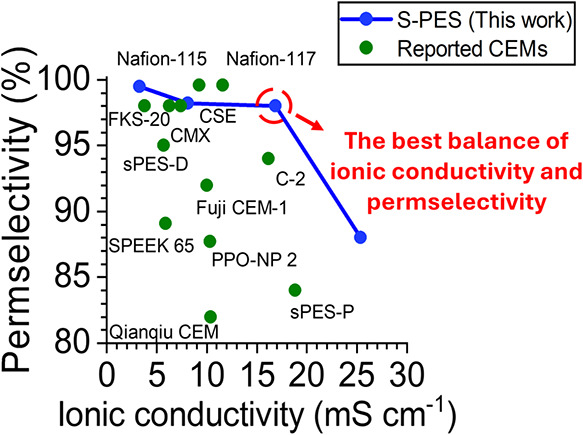

## 1 Introduction

Ion exchange membranes (IEMs) play a significant role in the advancement of numerous applications, including water treatment, industrial separation, and power generation, with a focus on selective ion dialysis, electrodialysis (ED), reverse electrodialysis (RED), and fuel cells.^1–3^ The performance of ion exchange membranes (IEMs) is generally governed by their physical and electrochemical properties, such as their area resistance (AR), permselectivity (α), ion exchange capacity (IEC), water uptake (WU) or swelling degree (SD), thickness (*L*), and fixed charge density (*C*_fix_). The majority of commercially available IEMs are manufactured as homogeneous membranes by using functionalized polymeric materials. These commercial membranes exhibit an AR ranging from 1 to 5 Ω cm^2^, an α above 90%, an IEC between 1.1 and 2.5 mequiv g^–1^, a WU up to 30%, and a thickness varying from 30 to 200 μm.^4–6^ As shown later in a comprehensive comparison of many reported membranes, Nafion-117 remains the best among the commercial membranes with 100% permselectivity and ionic conductivity of 11.6 mS cm^–1^ (1.73 Ω cm^2^). It is challenging to improve all of these properties concomitantly due to a trade-off paradox: all of these properties are strongly related and sometimes undermine each other. For example, increasing the functional groups in the polymer backbone may make it possible to produce IEMs with high ion exchange capacity and improved ionic conductivity; however, these membranes may also have low permselectivity, poor mechanical stability, low fixed charge density, and high swelling degree.^7,8^ Therefore, it is worthwhile to investigate and understand the interplay between these opposing characteristics via optimizing the degree of functionalization to develop appropriate ion exchange membranes with outstanding performance.

Among the polymer materials used for IEM fabrication, S-PES is a highly promising membrane material because of its chemical and thermal durability, exceptional mechanical properties resulting from its aromatic backbone, high ionic conductivity facilitated by the high concentration of sulfonic acid groups (which could be controlled by reaction conditions), and cost efficiency.^9–12^ Generally, S-PES can be produced by pre- or postsulfonation of commercially available PES. Owing to its low cost and ease of application, postsulfonation is more frequently employed than presulfonation. Thin membranes can be especially attractive since the decrease in membrane thickness can lead to a decrease in membrane resistance, giving rise to improved performance and reduced materials cost.

To the best of our knowledge, despite the wide literature existing on S-PES as a CEM, the crucial interplay between different polymer structures and the effect on the membrane’s ionic conductivity (or permselectivity) has not been explored. Insufficient sulfonation can restrict the overall ionic conductivity, while excessive sulfonation can bring on low membrane performance, as previously mentioned.^13^ CEMs of S-PES with different DS were previously reported by Klaysom et al.,^8^ who controlled DS by varying the reagent ratios. They achieved a high ionic conductivity of 29.7 mS cm^–1^ but with a very low permselectivity of 13.6%. When they achieved a high permselectivity of 95.2%, the ionic conductivity was only 0.06 mS cm^–1^. Avci et al.^6^ fabricated two S-PES membranes (sPES-D and sPES-P) using commercial S-PES (Konishi Co., Japan). The reported ionic conductivities were 5.73 and 18.86 mS cm^–1^ with a permselectivity of 95.0 and 84.0%, respectively. The high ionic conductivity has not been achieved without lowering the permselectivity. Cassady et al.^14^ and Rochow et al.^15^ used commercial S-PES (YANJIN Technology, Tianjin, China) with DS from 20 to 60% and reported high permselectivity values (91.1–100%). Membrane with DS of 50% was found to have an ionic conductivity of 3.13 mS cm^–1^ (area resistance = 0.67 Ω cm^2^), with a permselectivity of 95.4%. Komuta et al.^16^ have reported a series of S-PES membranes for dialysis applications. The best achieved balance between ionic conductivity and permselectivity was 3.66 mS cm^–1^ (AR = 1.03 Ω cm^2^) and 98.0%. Furthermore, Rezayani et al.^17^ extensively performed molecular dynamics (MD) simulations on S-PES membranes with various DS values to correlate some membrane morphological parameters, such as normalized water cluster size, pore limiting diameter (PLD), and water residence time, to the water/ion diffusion behavior.

All of these previous studies on S-PES as a CEM have not reported an optimal DS range; that is, the best balance between ionic conductivity and permselectivity was not achieved. Investigating the optimal DS value for S-PES is not only a modification of the same structure but also a regulation of several key morphological parameters that affect water and charge transfer through the membrane.

Therefore, in this work, we addressed the fine-tuning in DS for S-PES as CEMs by controlling the reaction time. We investigated the trade-off between ionic conductivity, permselectivity, and other membrane physical properties to find the optimal DS and the best performance as a CEM. This could open more prospects and insights for various membrane-based technologies, such as water desalination by ED, sustainable energy production using RED, and fuel cells.

## 2 Experimental Section

### 2.1 Materials

Poly­(ether sulfone) (SumikaExcel PES 7600P) was purchased from Sumitomo Chemical Company (Tokyo, Japan) and dried at 90 °C under vacuum for 24 h before use. 1-Methyl-2-pyrrolidinone (NMP; CAS No. 872–50–4, 99%), chlorosulfonic acid (CAS No. 7790–94–5, 97%), sulfolane (CAS No. 126–33–0, 95%) that was dehydrated using molecular sieves for 24 h before use (4A, Sigma-Aldrich Co.), analytical grade sodium chloride (CAS No. 7647–14–5), sodium hydroxide (CAS No. 1310–73–2), potassium chloride (CAS No. 7447–40–7), and phenolphthalein (CAS No. 77–09–8) were all purchased from FUJIFILM Wako Pure Chemical Corporation (Osaka, Japan). CSE Neosepta was purchased from ASTOM Corp., Japan.

### 2.2 Sulfonation Reaction

10.6 g of PES was stirred with 60 g of sulfolane in a three-neck flask until complete dissolution. 15.6 g of chlorosulfonic acid was added dropwise over 30 min. Then, the reaction mixture was refluxed under a nitrogen atmosphere at 100 °C in an oil bath.^18^ Sampling was done every 2 h up to 24 h, and the solution was poured into deionized water. The precipitated polymer pellets were washed with deionized water several times until a neutral pH was reached and dried at 70 °C under vacuum for 24 h. This drying process was repeated each time prior to use.

### 2.3 Membrane Preparation

The dried S-PES pellets were dissolved in NMP to form a 5% (w/v) polymer solution, and then, a suitable amount of the polymer solution was cast onto a glass surface. The S-PES membrane was obtained by drying the casting solution at 70 °C for 2 days in a vacuum oven. The membrane adsorbs water slowly, so it was further dried at 70 °C for 24 h in a vacuum oven before characterization.

### 2.4 Characterization of S-PES

#### 2.4.1 FT-IR

FT-IR spectra were recorded on dried S-PES and PES membrane samples by using a JASCO FT-IR-6200 spectrophotometer equipped with an ATR accessory (ATR PRO450-S, JASCO, Japan). Measurements were done with a resolution of 4 cm^–1^, and 32 scans were collected and averaged. Nitrogen gas purge was performed before the analysis.

#### 2.4.2 Scanning Electron Microscopy (SEM) and Elemental Analysis

Scanning electron microscopy images were recorded using an SEM SU5000 apparatus (Hitachi, Japan). Sulfur content was determined through energy-dispersive X-ray spectroscopy (EDX) by means of an EDX probe (AMETEX-30491, USA). DS values were calculated according to the following (eq 1)­

1 $$\mathrm{DS}\left(\%\right)=\frac{M-T}{H}\times 100$$

where *M* is the measured S/C atomic% in the S-PES sample, *T* is the theoretical S/C atomic% in PES (8.33%), and *H* is the maximum increase in the S/C% in the case of 100% DS (also 8.33%). Here, we assume one sulfonic acid group per monomeric unit for a full (100%) substitution.

#### 2.4.3 ^1^H NMR

Proton nuclear magnetic resonance (^1^H NMR) spectra were obtained using a JEOL AL 400 (400 MHz, 9.38T) apparatus with deuterated dimethyl sulfoxide (DMSO-*d*_6_) as the solvent. All samples were dried overnight at 60 °C under a vacuum before the measurement. The samples were dissolved in DMSO-*d*_6_ under an Ar atmosphere. All of the data are given as chemical shifts in δ (ppm) relative to (CH_3_)_4_Si.

DS of S-PES was calculated based on the integral peak values of the obtained spectra using the following (eqs 2,3)^19^

2 $$z=\frac{AH_{E}}{\sum {\mathrm{AH}}_{A,B,C,D}}$$

3 $$\mathrm{DS}\left(\%\right)=\frac{8z}{1+2z}\times 100$$

where AH_E_ is the integral peak value for H_E_, and ∑AH_A,B,C,D_ is the integral for H_A_, H_B_, H_C_, and H_D_ (see Figure S3).

#### 2.4.4 Thermal Gravimetric Analysis (TGA)

The thermal stability of PES and S-PES was investigated using a thermal gravimetric analysis apparatus (STA200RV, Hitachi, Japan) under a nitrogen gas flow of 200 mL min^–1^ with a heating rate of 10 °C min^–1^ in the temperature range of 25–600 °C. A 10 mg sample was dried at 60 °C under vacuum for each measurement.

### 2.5 Ion Exchange Capacity

Ion exchange capacity (IEC) is defined as milliequivalents of sulfonic acid groups per gram of dried membrane. IEC was determined by acid–base titration using 0.01 M NaOH. In the first step, the membrane sample was immersed in 1.0 M HCl for 48 h to ensure that all the negatively charged sulfonic acid groups were saturated with protons. Then, the membrane was rinsed with deionized water and immersed in a 2.0 M NaCl solution for 48 h to replace all protons with sodium ions. This step was repeated two times to ensure that all of the protons within the membrane matrix were released into the solution. Finally, all of the NaCl solutions were collected and titrated against 0.01 M NaOH using a phenolphthalein indicator. IEC was calculated using the following (eq 4)­

$$\mathrm{IEC}\left(\mathrm{meq}{\;g}^{-1}\right)=\frac{C\times V}{w_{\mathrm{dry}}}$$ 4

where *C* (mM) is the concentration of NaOH solution (0.01 M), *V* (L) is its volume consumed during titration, and *w*_dry_ (g) is the mass of the dry membrane.

DS is defined as the number of sulfonic acid groups per repeating unit of the polymer. It was calculated from the IEC value using the following (eq 5)^20,21^

5 $$\mathrm{DS}\left(\%\right)=\frac{[\left(\frac{\mathrm{IEC}}{1000}\right)\times M_{\mathrm{wt}}\left(\mathrm{RU}\right)]}{1-[\left(\frac{\mathrm{IEC}}{1000}\right)\times M_{\mathrm{wt}}\left(SO_{3}\right)]}\times 100$$

where *M*_wt_ (RU) and *M*_wt_ (SO_3_) are the molecular weights of the repeating unit and the pendant SO_3_ group with values of 232 and 81 g mol^–1^, respectively.

### 2.6 Water Uptake

Water uptake (WU) is defined as the weight of absorbed water in grams per gram of dried membrane. To calculate WU, each membrane was dried for 24 h at 70 °C in a vacuum oven and then immersed in deionized water for 48 h at room temperature. WU was calculated according to the following (eq 6)­

6 $$\mathrm{WU}\left(\%\right)=\frac{w_{\mathrm{wet}}-w_{\mathrm{dry}}}{w_{\mathrm{dry}}}\times 100$$

where *w*_wet_ and *w*_dry_ are the masses of the wet and dried membranes in grams, respectively.

### 2.7 Hydration Number

Hydration number (λ) is defined as the number of water molecules per ionic sulfonate group, and can be calculated according to the following (eq 7)^21^

7 $$\lambda =\frac{1000\left(w_{\mathrm{wet}}-w_{\mathrm{dry}}\right)}{18\times w_{\mathrm{dry}}\times \mathrm{IEC}}$$

### 2.8 Fixed Charge Density

Fixed charge density (*C*_fix_) is defined as mmol (or meq) of fixed charged groups per gram of absorbed water, and was calculated using IEC and WU values according to the following (eq 8)^7,22^

$$C_{\mathrm{fix}}\left(\mathrm{meq}\;g_{\mathrm{water}}^{-1}\right)=\frac{\mathrm{IEC}}{\mathrm{WU}\%}\times 100$$ 8

### 2.9 Contact Angle

The contact angle (CA) of water on PES and S-PES membranes was measured at room temperature using a contact angle measurement instrument (DMe-211 Plus; FAMAS software, Kyowa, Osaka, Japan). Five angles were measured in different places on the membrane surface, and the average value was taken.

### 2.10 Mechanical Strength

A tensile tester (Autograph AGS-X, Shimadzu, Japan) was used to investigate the mechanical strength of the dry and wet membranes at room temperature. The PES and S-PES (with different DS) membrane samples were pulled at 1.0 mm min^–1^ crosshead speed. Membranes with dimensions of 15 mm × 10 mm and a thickness of 0.07 mm were prepared for the tensile test. Samples were dried at 70 °C for 24 h under vacuum for the dry state measurements and then immersed in deionized water for 24 h for the wet state measurements. The membrane thicknesses were measured using a Niigata Seiki SK Micrometer Stand S-Type 25–100 MS-SG, Japan.

### 2.11 Ionic Conductivity Measurement

The membrane resistance was determined by applying an alternating current (AC) at a frequency of 10 kHz to a two-compartment cell filled with 0.5 M NaCl aqueous solution,^22,23^ as shown in Figure 1a, using an LCR meter (DE-5000, Taiwan). Each membrane was immersed in 0.5 M NaCl for 24 h before measurement. The solution was circulated using an external feed pump with a flow rate of 100 mL min^–1^ at 25 ± 0.1 °C. The two compartments were connected to the LCR meter with two fixed platinum electrodes that remained in the same position for all measurements. The membrane resistance (*R*_m_, Ω) was calculated by subtracting the resistance of the blank (no membrane present). The area resistance can be obtained by dividing *R*_m_ by the effective area of the membrane (*A*, 0.949 cm^2^). The membrane ionic conductivity (σ, S cm^–1^) was calculated using the following (eq 9)­

9 $$\sigma \left(S\;{cm}^{-1}\right)=\frac{L}{R_{m}A}$$

where *L* is the thickness of the membrane (cm).

**Figure 1:**
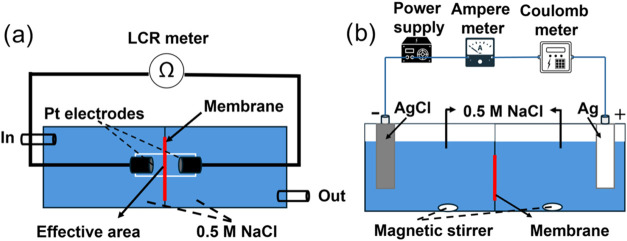
Schematic diagram of the measurement setup for determining membrane ionic conductivity (a) and permselectivity (b).

### 2.12 Permselectivity Measurement (Hittorf Method)

The membrane permselectivity was determined by means of the dynamic state ion transport number (*t*_d*+*_) as an indicator of the counterion permselectivity for IEMs.^23,24^ Therefore, the permselectivity term (α) will be used in our study to refer to the counterion selective permeation through the membrane. The measurement was carried out using a two-chamber cell, as shown in Figure 1b. A direct current with a current density of 10 mA cm^–2^ was applied between the two Ag and AgCl electrodes in a two-chamber cell containing 0.5 M NaCl at 25 °C.^22^ After the experiment, the conductivity difference between the two chambers was measured to determine the corresponding concentration change caused by ion transport via the membrane. The following equation was used to determine the dynamic state ion transport number (eq 10)­

10 $$\alpha \left(\%\right)=t_{d+}=\frac{\Delta m\times V\times F}{Q}\times 100$$

where Δ*m*, *V*, *F*, and *Q* are the equivalent change in ions, solution volume (*L*), Faraday constant (96485 C mol^–1^), and electric charge (*C*) flowing across the membrane during the test, respectively.

## 3 Results and Discussion

### 3.1 Sulfonation Reaction

Sulfonation of PES is basically a second-order bimolecular aromatic electrophilic substitution reaction (PES + ClSO_3_H → S-PES + HCl) that obeys the second-order rate law according to the following (eq 11).^25,26^

11 $$\frac{dc}{dt}=k\left(a-c\right)\left(b-c\right)$$

where *a* and *b* are the initial concentrations of PES and chlorosulfonic acid (M), respectively; *c* is the concentration of produced S-PES at time *t*; and *k* is the reaction rate constant (M^–1^ h^–1^). From eq 11, the following equation is obtained (eq 12).^25^

12 $$\frac{1}{a-b}\ln\frac{b\left(a-c\right)}{a\left(b-c\right)}=\mathrm{kt}$$

The reaction rate (followed by the change in DS with time) was found to obey eq 12, with a correlation coefficient of 0.992, as shown in Figure S1a. The rate constant was calculated to be 2.48 × 1 × 10^–2^ M^–1^ h^–1^.

Generally, electron-withdrawing groups deactivate the aromatic ring for sulfonation. Since the sulfone linkage in PES is an electron-withdrawing substituent, the sulfonation reaction was regarded to take place only at the meta position to the sulfone linkage, that is, the ortho position to the ether linkage in PES. As previously reported by Hu et al.,^27^ who performed a molecular simulation for the sulfonation site during similar reaction, indicating the feasibility of the attack on the meta position with respect to the sulfone linkage due to the lower activation energy of its transition state while the other sites adjacent to the bulky sulfone linkage is hindered by steric repulsion and hence have a higher activation energy. The introduction of the strong electron-withdrawing sulfonic acid group further deactivates the aromatic ring toward sulfonation. Thus, on average, one sulfonic acid group per monomeric unit can usually be introduced into the PES backbone at position meta to the sulfone group adjacent to the ether linkage, as we show in Figure 2a.^28^ Therefore, PES with only one of the protons substituted by the sulfonic acid group was considered the product.

**Figure 2:**
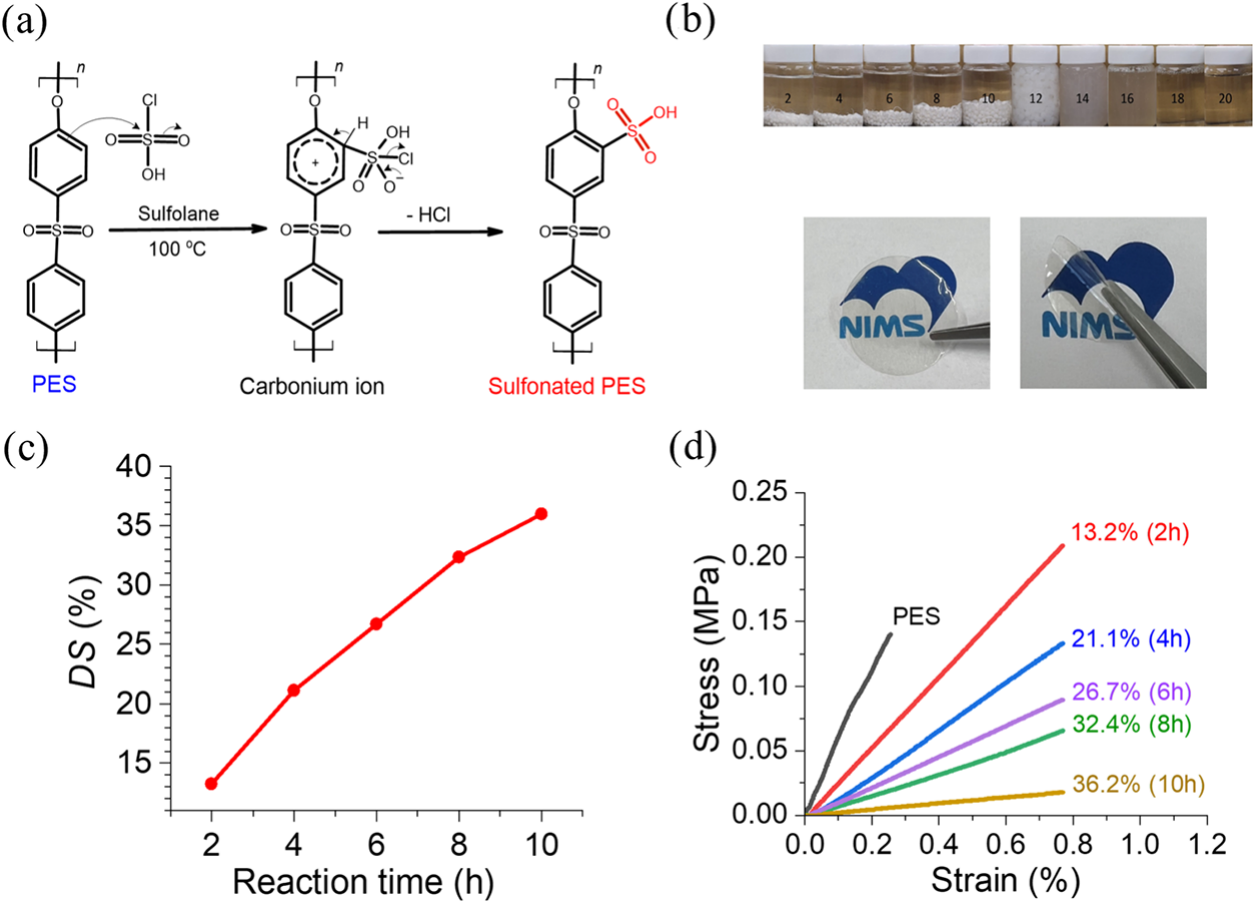
(a) Sulfonation reaction mechanism for PES, (b-top) appearance of the precipitated S-PES pellets at different reaction times/hour, after immersion in deionized water for 7 days, (b-bottom) appearance of the flat and bent S-PES membranes with a DS of 36.2%, (c) DS of the produced S-PES at different reaction times, (d) stress–strain curves for PES and S-PES membranes with different DS in the dry state.

DS values for the produced S-PES were calculated based on three techniques: (a) measuring the percentage of sulfur in a dry sample using EDX elemental analysis according to eq 1, (b) using the integral peak values in the ^1^H NMR spectra based on eqs 2 and[Disp-formula eq3], and (c) using IEC values calculated from the titration experiment according to eq 5. The values of DS using the three techniques, as well as the average values as a function of the reaction time, are shown in Table S1 and Figure 2c. Since the three methods have a certain experimental error, we used the mean average values without any weighing for the discussion on DS.

When the reaction time was 2–10 h, similar precipitated pellets were formed. However, the pellets produced by longer reaction times tend to swell after being kept in deionized water. As shown in Figure 2b, the pellets produced by a 12 h reaction time were largely swollen in deionized water after immersion for 7 days. After 14 days of water immersion, this sample completely dissolved in deionized water, as well as other samples produced in longer reaction times (Figure S1b). Probably, S-PES with a high DS value behaves as a polymer surfactant and slowly forms a colloidal dispersion in deionized water.

The produced S-PES membranes have been characterized using SEM (Figure S2), FT-IR (Figure 4a), ^1^H NMR (Figure S3), and TGA-DTA (Figure S4) to confirm the membrane morphology, chemical structure, and thermal stability, respectively.

### 3.2 Physical and Electrochemical Properties of S-PES Membranes

#### 3.2.1 Basic Physical Properties

IEC, WU, *C*_fix_, and *CA* are crucial parameters ultimately governing the ionic conductivity and permselectivity of IEMs. These basic physical properties are summarized in Table 1 as a function of the reaction time and DS. IEC and WU monotonously increase with increasing DS, with a concomitant decrease in *C*_fix_, in line with general expectations. As the number of SO_3_H groups increases, the membranes become more hydrophilic. This trend can be confirmed by the decreasing water contact angle from 95.6° for PES to 50.5° for S-PES after 10 h of reaction time (Figure 3a). The hydration number was also found to be increasing with DS due to the increased largest cavity diameter (LCD), giving a larger space for more water molecules to surround the sulfonate groups.^17^ The IEC value in the case of DS = 0% (for PES) is due to the fact that the membrane itself absorbed a small amount of the solutions during the titration experiment, and not due to the presence of any ion exchange groups. So, this value can be neglected.

**Figure 3:**
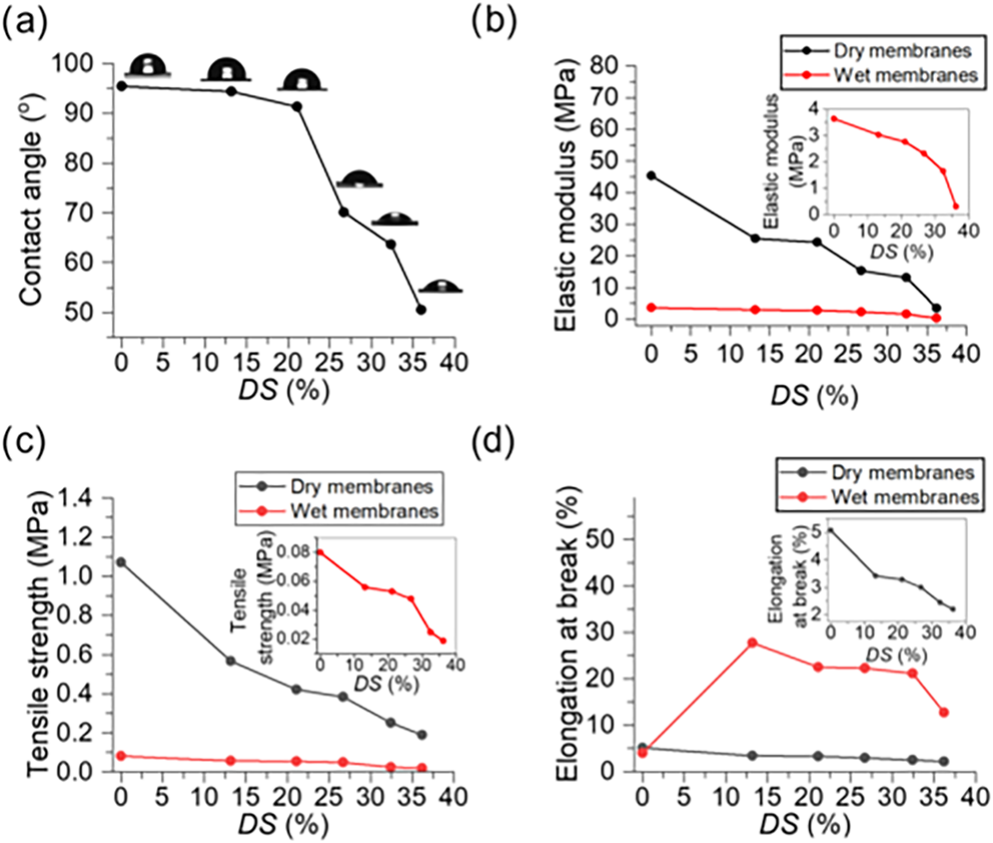
(a) Water contact angles of PES and S-PES membranes with different DS, (b) elastic moduli of the dry and wet PES and S-PES membranes with different DS (inset shows the elastic modulus of the wet membranes), (c) tensile strength of the dry and wet PES and S-PES membranes with different DS (inset shows the tensile strength of the wet membranes), (d) elongation at break of the dry and wet PES and S-PES membranes with different DS (inset shows the elongation at break of the dry membranes).

**Table 1:** Basic Properties of the Fabricated PES and S-PES Membranes as a Function of the Reaction Time and DS

reaction time (h)	DS (%)	IEC (meq g^–1^)	WU (%)	*C*_fix_ (meq g_water_^–1^)	CA(°)	λ
0	0	0.08	1.2	6.8 ± 1.0	95.6	0
2	13.2	0.61	5.7	10.8 ± 0.8	94.4	5
4	21.1	0.90	10.0	9.0 ± 0.1	91.3	6
6	26.7	1.03	16.1	6.4 ± 0.7	70.1	9
8	32.4	1.29	20.6	6.3 ± 0.4	63.7	9
10	36.2	1.36	26.3	5.2 ± 0.3	50.5	11

#### 3.2.2 Mechanical Strength

Because the fabricated S-PESs are targeted to be used in membrane-based technologies, especially for ED and RED, investigating the mechanical properties in both dry and wet states is imperative. In the dry state, the mechanical strength and elongation at break decrease for the S-PES membranes compared with the pure PES membrane (Figure 3c and d). This may be explained by the random introduction of the highly polar sulfonic acid groups into the PES chain, generating inhomogeneities along the polymer backbone. The configuration of the polymer chains also expands with the introduction of sulfonic acid groups, hindering entanglement formation and weakening primary intermolecular interactions (such as π stacking).^29^ As shown in Figure 3c and d, the wet S-PES membranes exhibit a much lower tensile strength and higher elongation at break (by around 0.9 and 6 times, respectively) compared to dry ones. The polymeric chains are plasticized by the absorbed water molecules, resulting in an increase in their segmental mobility, significantly affecting their mechanical response.^29^ Moreover, the elongation of the wet S-PES membranes decreases with increasing DS, as in the case of higher DS, the membranes are more swollen due to the higher water uptake, and further elongation will be more difficult. The elastic modulus of PES and S-PES membranes at the dry and wet state (Figure 3b) was calculated from the stress–strain curves shown in Figures 2d andS5a, respectively.

#### 3.2.3 Long-Term Stability

The membrane’s long-term and thermal stabilities were confirmed using FT-IR, as shown in Figure 4, in which the S-PES membrane sample (DS = 36.2%, 10 h reaction time) was immersed in 0.5 M NaCl at room temperature for more than 120 days, then rinsed with deionized water and dried at 60 °C for 24 h before the measurement (Figure 4a). Additionally, the stability of S-PES membranes with different DS values in 0.5 M NaCl at 50 °C for 14 days was tested, as shown in Figure 4b. Neither membrane was dissolved nor was it swollen after the experiment. These results confirm the stability of the membrane at 25 and 50 °C. The intensity of the characteristic peak for the SO_3_H group at 1025 cm^–1^ increases with reaction time. We also confirmed that no change in these peaks was observed after immersion at 50 °C for 14 days. The surface and cross-sectional photo images of the S-PES membrane in Figure 4c (DS = 36.2%, 10 h reaction time) confirm that no surface morphological change occurs after stirring in 0.5 M NaCl for 14 days at 50 °C. When the temperature was raised to 70 °C, this membrane dissolved. S-PES membrane with DS of 32.4% (8 h reaction time) also dissolved in the same condition, indicating the maximum possible operating temperature at high DS values (above 30%) is less than 50 °C. S-PES membrane with DS of 27.1% (6 h reaction time) was still stable at 70 °C. Note that the S-PES membrane (8 h) is slightly swollen in a 10% methanol solution; these membranes are not stable in water/alcohol mixtures.

**Figure 4:**
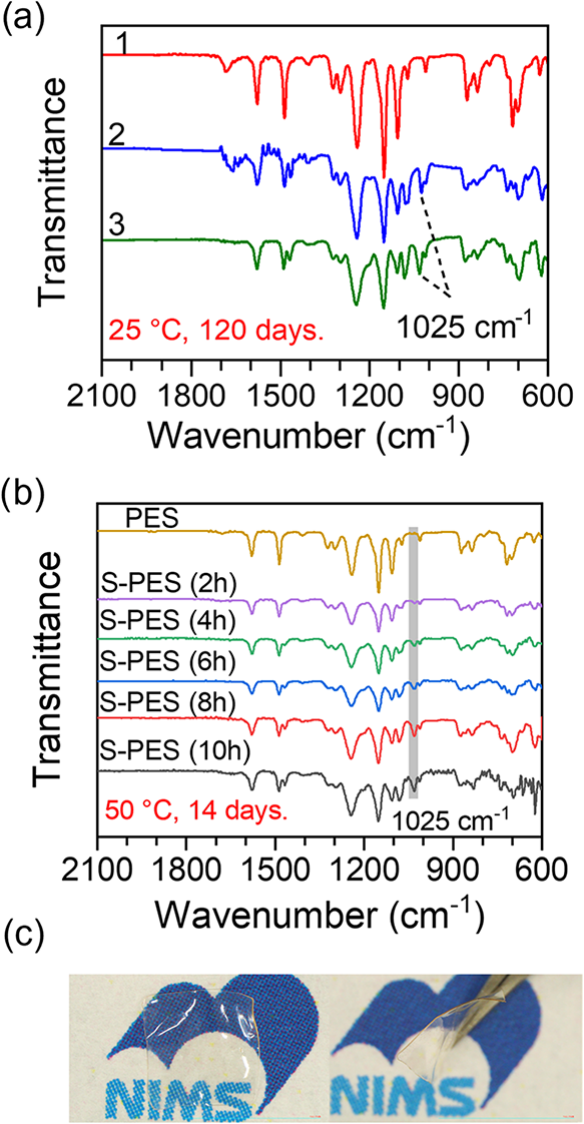
(a) FT-IR spectra for (1) PES, (2) pristine S-PES, and (3) S-PES after immersion in 0.5 M NaCl for 120 days, (b) FT-IR spectra for PES and S-PES produced at different reaction times after stirring in 0.5 M NaCl for 14 days at 50 °C, (c) surface and cross-sectional images of S-PES membrane (DS = 36.2%) after stirring in 0.5 M NaCl for 14 days at 50 °C.

#### 3.2.4 Membrane Ionic Conductivity

The membrane ionic conductivity is one of the principal parameters for the performance of the IEMs. Generally, the presence of ion exchange groups within the membrane matrix is one of the main factors that determines the WU and ionic conductivity of IEMs. It is expected that with increasing IEC, the WU and membrane ionic conductivities will concomitantly increase. In addition to IEC, there are some factors affecting WU that have to be taken into consideration, such as polymer hydrophilicity and membrane morphology.^30–32^ The right balance between all of these parameters is very important to achieve an outstanding performance.^4^

The ionic conductivity of the fabricated S-PES membranes as a function of DS and reaction time is shown in Figure 5a. The corresponding area resistance values derived from the same experiments are shown in Figure S5b. The membrane ionic conductivity increases up to 25.34 mS cm^–1^ with increasing DS to 36.2% (10 h reaction time). Rezayani et al. noted that when the hydration number was 10 for S-PES with DS of 30 and 40%, the water networks within the polymer matrix were almost interconnected, resulting in a high diffusion rate.^17^ At this stage, the pore limiting diameter (PLD) also started to exceed the diameter of the water molecule (0.32 nm). As the PLD exceeded the critical diameter with higher DS, it could be expected that both small cations and the large water molecule would permeate through the membrane and permselectivity would decrease. In our study, the hydration numbers were 9 and 11 for our prepared membranes at DS 32 and 36%, respectively. As we have shown, DS of 32% provided the best balance of ionic conductivity and permselectivity, which has a hydration number close to the reported value by Rezayani et al.

**Figure 5:**
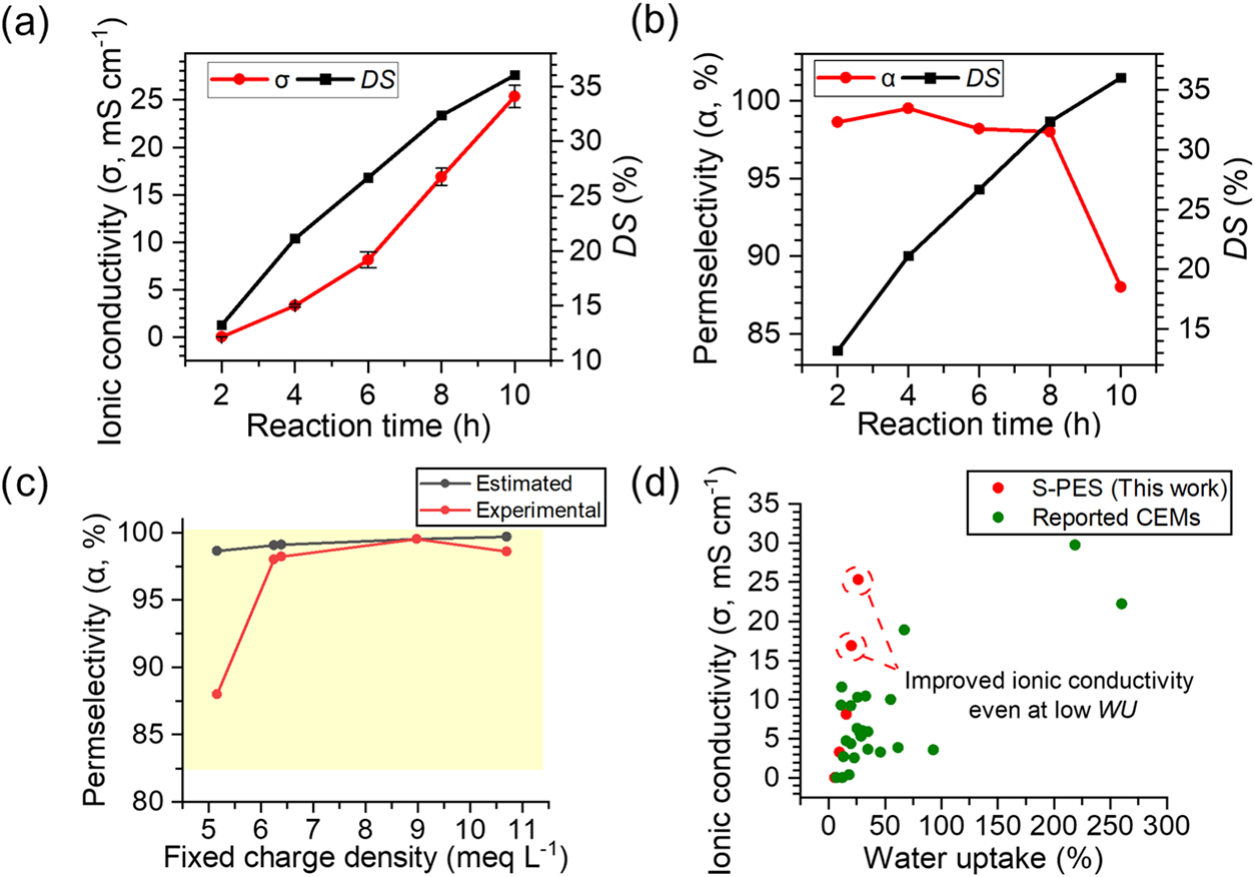
(a) Ionic conductivity and (b) permselectivity of the fabricated S-PES membranes as a function of reaction time, (c) estimated and experimental permselectivity of the different S-PES membranes as a function of the fixed charge density (yellow region is the permselectivity range of commercial CEMs in Table 2), (d) ionic conductivity of S-PES and previously reported CEMs as a function of water uptake.

#### 3.2.5 Membrane Permselectivity

The permselectivity of a membrane is defined as the flux of an individual element in comparison to the total flux through the membrane under a certain driving force.^7^ The permselectivity of IEMs is a measure of how well the membrane can distinguish between anions (such as Cl^–^) and cations (such as Na^+^).^7^ Similar to membrane ionic conductivity, membrane permselectivity is also an essential factor determining membrane performance.

Regarding permselectivity, *C*_fix_ exerts one of the most prominent impacts on this property.^6,7,22^ For example, a high IEC will not surely indicate high permselectivity, as the membrane may have a high WU, leading to a low *C*_fix_ and weaker exclusion of co-ions. As a result, the permselectivity value may be lower than expected. However, it is possible to produce membranes with a higher permselectivity by increasing IEC if the WU is well controlled.^33^

The permselectivity of the prepared S-PES membranes as a function of the reaction time is shown in Figure 5b. As mentioned before, the higher the *C*_fix_, the stronger the exclusion of co-ions and the higher the permselectivity. As presented in Table 1, there is a larger increase in WU with increasing DS compared to the IEC values. Therefore, *C*_fix_ decreases with time in line with eq 8. Thus, it can be noticed that for the higher *C*_fix_ values (10.8–6.3 mequiv g_water_^–1^), the S-PES membranes show comparable permselectivity values between 98.0 and 99.0%. At a *C*_fix_ of 5.2 mequiv g_water_^–1^, the permselectivity of the membrane decreases to 88.0%. This can give an indication of the boundary line where the decrease in *C*_fix_ results in a significant decline in permselectivity for S-PES membranes.

Also, another reason for the decreasing permselectivity lies in the increased WU, providing higher ionic mobilities for the co-ions due to higher free water content. An increased volume of free water within the membrane can result in greater space for co-ions to pass through the membrane with less friction between the ions and the membrane polymer, as well as among the ions themselves (solute-membrane and solute–solute friction).^34^

The permselectivity of the counterion (Na^+^ in the case of NaCl solution) through the ion exchange membrane can be theoretically estimated using the following equation^22^

13 $$e\mathrm{stimated permselectivity}=\frac{{ú}_{\mathrm{Na}}\left[\left(\sqrt{{C_{\mathrm{fix}}}^{2}+4C^{2}}\right)+C_{\mathrm{fix}}\right]}{{ú}_{\mathrm{Na}}\left[\left(\sqrt{{C_{\mathrm{fix}}}^{2}+4C^{2}}\right)+C_{\mathrm{fix}}\right]+{ú}_{\mathrm{Cl}}\left[\left(\sqrt{{C_{\mathrm{fix}}}^{2}+4C^{2}}\right)-C_{\mathrm{fix}}\right]}$$

where *ú*_Na_ and *ú*_Cl_ are the ionic mobilities of Na^+^ and Cl^–^, respectively (*ú*_Na_/*ú*_Cl_ = 0.656).^14,35^ *C* is the concentration of the NaCl solution (0.5 mol L^–1^).

Using the above equation, the permselectivity values for S-PES membranes with different DS can be theoretically estimated as a function of *C*_fix_ and compared to the experimental ones as presented in Figure 5c. As concluded from the figure, the experimental and theoretical values are in strong agreement with each other, ranging from 98.0 to 99.6%, except for the membrane with the lowest *C*_fix_ of 5.2 mequiv g_water_^–1^, which has a DS of 36.2%. This may be explained by the heterogeneity of the fixed ion concentration at high WU compared to the other S-PES membranes with lower DS.^22,36^

#### 3.2.6 Trade-Off between Ionic Conductivity and Permselectivity

A detailed understanding of the complex interplay among IEC, WU, and *C*_fix_ is crucial for the development of an IEM with high ionic conductivity and permselectivity, as discussed above. Table 2 shows a comparison between S-PES membranes in this work and various CEMs reported in the literature. One of the outstanding merits of the S-PES membranes in this study is the high ionic conductivity without absorbing too much water and thus, without compromising the permselectivity, compared to various reported CEMs, as shown in Figures 5d and S6b. As a result, the essential balance between ionic conductivity and permselectivity is achieved.

**Table 2:** Comparison between the Produced S-PES Membranes in This Work and Other CEMs Reported in the Literature

membrane	*L* (μm)	IEC (meq g^–1^)	WU (%)	*C*_fix_ (meq g_water_^–1^)	α (%)	AR^a^ (Ω cm^2^)	σ^a^ (mS cm^–1^)	refs
S-PES (10 h)	70 ± 10	1.36	26.3	5.2	88.0	0.41	25.34	Ours
S-PES (8 h)	70 ± 10	1.29	20.6	6.3	98.0	0.49	16.85	Ours
S-PES (6 h)	70 ± 10	1.03	16.1	6.4	98.2	0.65	8.14	Ours
S-PES (4 h)	70 ± 10	0.90	9.99	9.0	99.5	2.10	3.31	Ours
S-PES (2 h)	70 ± 10	0.61	5.67	10.8	98.6	344.50	0.02	Ours
CSE	150	-	-	-	98.0	2.14	7.15	Ours
sPES-25 (E)	80 ± 10	0.53	7.26	7.28	88.8	278.74	0.03	^8^
sPES-40 (E)	80 ± 10	1.44	12.5	11.45	95.2	135.36	0.06	^8^
sPES-55 (E)	80 ± 10	1.58	18.2	8.66	80.5	18.88	0.42	^8^
sPES-25 (P)	80 ± 10	0.73	260	0.28	13.7	0.36	22.22	^8^
sPES-40 (P)	80 ± 10	0.60	219	0.27	13.6	0.27	29.70	^8^
sPES-P^b^	83 ± 6	1.15	67.2	1.70	84.0	0.44	18.86	^6^
sPES-D^b^	63 ± 6	1.19	28.0	4.30	95.0	1.10	5.73	^6^
SPES-20^b^	-	0.92	-	-	100.0	-	-	^14^
SPES-30^b^	-	1.34	-	-	99.9	-	-	^14^
SPES-40^b^	-	1.72	-	-	98.1	-	-	^14^
SPES-50^b^	21	2.08	-	-	95.4	0.67	3.13	^14,15^
SPES-60^b^	-	2.42	-	-	91.1	-	-	^14^
SPES-CEM 1	34	0.98	13.0	1.84	97.0	1.03	3.30	^16^
SPES-CEM 6	63	1.69	7.0	1.77	98.0	1.72	3.66	^16^
Nafion-117	201 ± 8	0.90	11.7	7.70	100	1.73	11.61	^44^
Nafion-115	139 ± 8	0.90	11.2	8.00	100	1.50	9.26	^44^
Fuji CEM-1	120	1.96	55.0	3.60	92.0	1.20	10.00	^6^
Fuji CEM10	-	1.70	21.0	8.00	94.7	2.30	-	^45^
CIMS	150	2.30	30.0	-	98.0	2.49	6.02	^37,46^
CMX	170	-	-	1.86	98.0	2.70	6.29	^37^
FKS-20	18	-	-	1.93	98.0	0.47	3.83	^37^
C-2	34	-	-	-	94.0	0.21	16.19	^37^
Fumasep FKD	113	1.14	29.0	-	89.5	2.14	5.28	^4,47^
Fumasep FKS	40	1.54	13.5	-	94.2	1.50	2.67	^7^
Asahi CMV	100	2.00	20.0	10.10	98.8	2.30	4.35	^7^
Asahi CSO	100	1.04	16.0	6.50	92.3	2.26	4.76	^1^
SPEEK 65	72	1.76	35.0	4.90	89.1	1.22	5.90	^7^
SPEEK 40	53	1.23	23.0	5.30	85.3	2.05	2.59	^7^
Qianqiu CEM	205	1.21	33.0	3.70	82.0	1.97	10.41	^7^
PPO-NP 1	100	1.0	20.0	5.00	84.4	1.09	9.17	^1^
PPO-NP 2	100	1.4	26.0	5.40	87.7	0.97	10.31	^1^
PPO–PVA 1	50	1.91	46.0	4.20	87.3	1.54	3.25	^1^
PPO–PVA 2	50	1.80	62.0	2.90	84.2	1.30	3.85	^1^
PPO–PVA 3	50	1.58	93.0	1.70	81.0	1.41	3.55	^1^

a Tested in 0.5 M NaCl.

b Casted from a commercial SPES powder.

It can be concluded that the fabricated membrane using S-PES with a DS value of 36.2% (10 h reaction time) shows the highest ionic conductivity value of 25.34 mS cm^–1^, and the lowest permselectivity value of 88.0% compared to the other S-PES membranes fabricated with shorter reaction times (2–8 h), whereas the membrane with a DS of 32.4% (8 h reaction time) provides a good balance in the trade-off between these properties, with a significantly high ionic conductivity of 16.85 mS cm^–1^ (an area resistance of 0.49 Ω cm^2^) and high permselectivity of 98.0%. Therefore, among the membranes fabricated in this work, we see S-PES with a DS of 32.4% (8 h reaction time) as a promising CEM with the best sulfonation-controlled trade-off between ionic conductivity and permselectivity, as compared to previous studies on S-PES that have been discussed in detail in the introduction section.

For direct experimental comparisons and performance validation, CSE, Neosepta (ASTOM Corp., Japan), has been used for side-by-side evaluation. Additionally, in our previous work by Sugimoto et al.,^37^ the performance evaluation of some leading commercial membranes, such as CMX, CIMS, and C-2 (ASTOM Corp., Japan), as well as FKS-20 (FUMATECH BWT GmbH, Germany), has been conducted using the same setups and under the same conditions as our S-PES membranes, so we also used these data for direct comparisons. Figure 6 shows the performances of the best reported membranes from Table 2, including those from the side-by-side evaluation. The best commercial membrane, Nafion-117, has 100% permselectivity and an 11.61 mS cm^–1^ ionic conductivity. Our best membrane with a DS of 32% has a comparable permselectivity of 98% but ionic conductivity is 45% higher than that of Nafion-117. Fuji CEM-1 has even lower permselectivity and ion conductivity of 92% and 10 mS cm^–1^, respectively.

**Figure 6:**
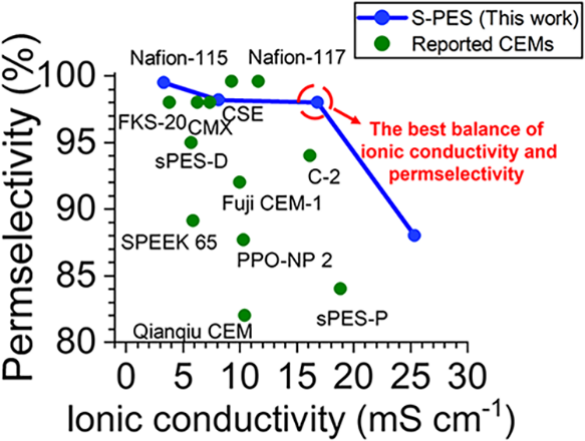
Ionic conductivity and permselectivity of S-PES membranes fabricated in this work compared to previously reported cation exchange membranes.

Avci et al. reported the performances of CEMs made of commercial S-PES (Konishi Co., Japan) with an IEC value of 1.19 mequiv g^–1^ and showed a power density output of 3.92 W/m^2^ for 0.1/4.0 M NaCl at 25 °C in RED.^6^ Therefore, the power density output of our optimum membrane may largely exceed this value, since the resistance of our membrane is just 45% of that of this membrane. Of course, this is the case in which CEM resistance is the major factor in the total resistance of the RED setup. In the practical application, it is not easy to achieve an output higher than 5.0 W/m^2^.

Although Nafion membranes have high performance, several drawbacks limit their future prospects. On the one hand, the high production cost due to the fluorination step makes Nafion an expensive option for various technologies. On the other hand, Nafion-117 is a typical perfluorinated sulfonic acid (PFSA) membrane, which can be a potential source of many perfluorinated chemicals when used and disposed of, including the hazardous and environmentally persistent perfluoro carboxylic acids (PFCAs).^38,39^ These chemicals are currently present in human blood and are omnipresent around the world.^40^ The presence of PFCAs in the environment has raised widespread concerns due to immunotoxicity, carcinogenicity, developmental, and hormonal impacts based on laboratory animal toxicology investigations.^41^ On the other hand, although PES-based membranes are degraded to microplastics in the long term, it is expected to decompose into harmless materials, being a better alternative from the environmental point of view.^42,43^ Therefore, these functional, economic, and environmental challenges have triggered research toward better alternatives.

## 4 Conclusion

In this work, a series of S-PES with various DS were successfully fabricated by sulfonation of the PES precursor using chlorosulfonic acid. The S-PES membranes were prepared by casting through the solvent evaporation method. The correlation between the physical and electrochemical properties of the fabricated S-PES membranes was investigated in detail with varying DS. There is a crucial interplay between various membrane properties that eventually drives the cation exchange performance. An increase in the number of introduced ionic groups (sulfonic acid groups) results in a more hydrophilic character, higher WU, higher IEC, and higher ionic conductivity. The produced S-PES membrane with an optimum DS of 32.4% has an outstanding ionic conductivity of 16.85 mS cm^–1^ and an almost ideal permselectivity of 98.0% (in 0.5 M NaCl). This membrane shows a significant improvement in the trade-off between ionic conductivity and permselectivity compared with previously reported S-PES membranes and commercial benchmark CEMs. Therefore, these fabricated S-PES membranes are promising CEMs, especially for the ED and RED.

## Supplementary Material



## Notes

Additional experimental data (DOC/PDF); sulfonation reaction kinetics; photographs of the precipitated S-PES pellets; calculated DS at different reaction times using different techniques; SEM images of S-PES membrane; FT-IR, ^1^H NMR, TGA, and DTA analysis for PES and S-PES; stress–strain curves of wet PES and S-PES membranes; area resistance and water uptake of the fabricated S-PES membranes as a function of reaction time and DS; and permselectivity of S-PES and previously reported CEMs as a function of water uptake (PDF)
