# Supplementary material for: Balanced Ionic Conductivity and Permselectivity of Cation Exchange Membranes Prepared from Sulfonated Poly(ether sulfone)
Source: ACS Omega. 2025 Dec 19;11(1):1437–46. doi: 10.1021/acsomega.5c09018 (PMC12809513; doi:10.1021/acsomega.5c09018)
Supplement: Supplementary file 1 [file ao5c09018_si_001.pdf]

Supporting Information

Balanced ionic conductivity and permselectivity of cation exchange membranes prepared from sulfonated poly(ether sulfone)

*Hussien K. Srour [a], Mizuki Inoue [a], Edhuan Ismail [a], Minato Higa [b], Mitsuru Higa [b], László Szabó [c], and Izumi Ichinose [a]**

[a] Research Center for Macromolecules and Biomaterials, National Institute for Materials Science, 1-1 Namiki, Tsukuba 305-0044, Japan

[b] Graduate School of Science and Technology for Innovation, Yamaguchi University, 2-16-1 Tokiwadai, Ube, Yamaguchi 755-8611, Japan

[c] Center for Advanced Materials, Forestry and Forest Products Research Institute, 1 Matsunosato, Tsukuba, Ibaraki 305-8687, Japan.

*Email: ICHINOSE.Izumi@nims.go.jp

Contents

1. **Sulfonation reaction kinetics and appearance of the produced S-PES pellets**.........................2

2. **Characterization of S-PES** 3

3. **Physical and electrochemical properties**..7

References...8

1. Sulfonation reaction kinetics and appearance of the produced S-PES pellets

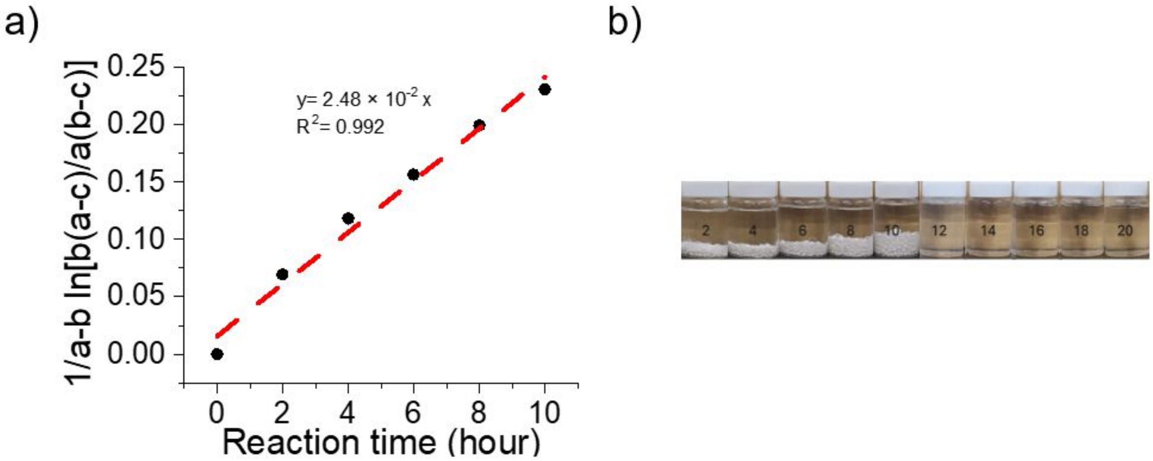

Figure S1. (a) Kinetics of PES sulfonation using chlorosulfonic acid, (b) appearance of the precipitated S-PES pellets at different reaction times/hour, after immersion in deionized water for 14 days.

Table S1. Calculated DS (%) at different reaction times.

Reaction time (h)	*DS*%			
	Titration	EDX	^{1}H-NMR	Average
2	14.9±1.9	13.8±1.2	10.6±0.4	13.2±3.5
4	22.5±1.2	20.1±1.0	20.7±0.7	21.1±2.9
6	26.1±1.4	28.9±1.5	25.1±1.2	26.7±4.1
8	33.4±0.7	29.6±1.2	34.1±0.9	32.4±2.8
10	35.5±1.1	34.0±1.8	39.2±0.6	36.2±3.6
12-24*	-	-	-	-

*Sample is completely dissolved in water.

While titration indicates the practical amount of the ionic exchangeable sites, it is carried out completely in a manual manner with a lot of steps, which gives a certain range of errors. EDX gives a complete survey of the exact elemental composition, which helps to calculate *DS* easily, but the peak overlapping of different elements causes an experimental error. ^{1}H-NMR is very structure-sensitive and is not affected too much by sample contamination, resulting in more accurate results for *DS*. However, it gives an error range at low *DS* values. From these reasons, we selected the mean average value of the three techniques.

2. Characterization of S-PES

2.1. SEM

After casting the produced S-PES from the NMP solution into membranes, the surface morphology as well as the cross-sectional structure were examined as shown in Figure S2. The membrane exhibited a smooth and dense structure without any visible microscale pores or defects, making the membranes mechanically durable with good barrier properties. The pictures of the fabricated S-PES membrane are shown in Fig. 2b.

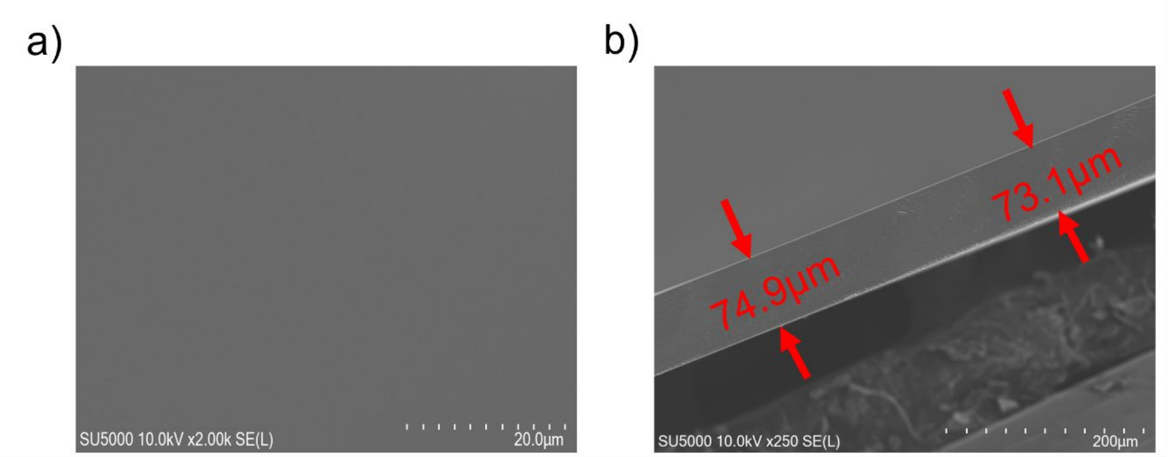

Figure S2. (a) Surface and (b) cross-sectional SEM images for S-PES membrane with a *DS* of 36.2%.

2.2. ^{1}H-NMR

The sulfonation of PES was confirmed by ^{1}H-NMR, as shown in Figure S3c and d. The spectrum of PES shows two major peaks originating from H_A and H_B protons at 8.0 ppm and 7.3 ppm, respectively. After sulfonation, new peaks (H_E, $H_{C,}$ and H_D) were observed at 8.3, 7.6, and 7.1 ppm, respectively. The assigned hydrogens for PES and S-PES are shown in Fig. S3a and b. The presence of the sulfonic acid group can be confirmed by the down-field shift of the signal at 8.0 to 8.3 ppm for the hydrogen located at the ortho position to the sulfonic acid group (H_E), in agreement with previous reports [2-4]. The ratio between the peak area of the signal for H_E and the peak areas of other aromatic hydrogens ($H_{A, B, C, D}$) was determined to calculate *DS* based on Eq. 2 and Eq. 3 shown in main body. For 10 h reaction time, the *DS* was found to be 39.2%, which was slightly higher than the calculated values from elemental analysis (34.0%) and titration (35.5%).

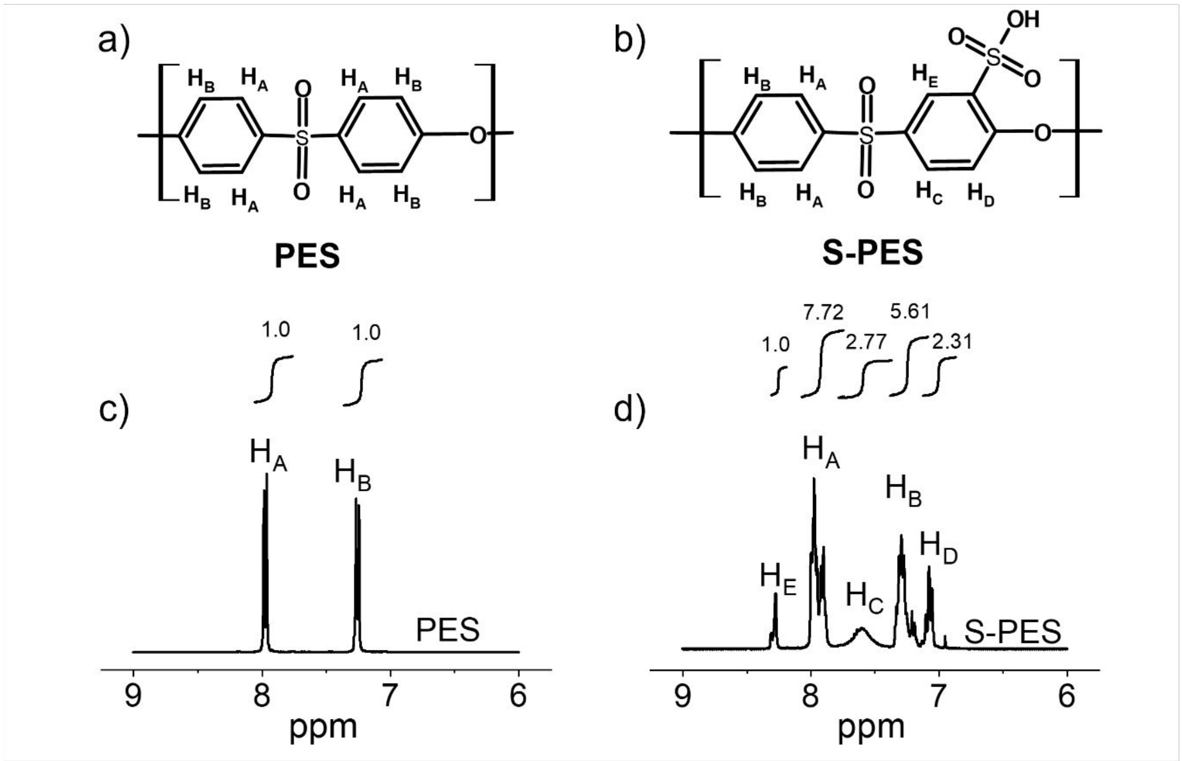

Figure S3. Assigned hydrogens for (a) PES and (b) S-PES, and 1H-NMR spectra with the integral peak values (c) for PES and (d) for S-PES. S-PES with an average *DS* of 36.2% was used for the analysis.

2.3. FT-IR

FT-IR spectra were recorded on PES and S-PES samples to verify the successful introduction of sulfonic acid groups (SO_3H) into the polymer chain after the sulfonation reaction (Figure S3a). The absorption peak at around 1025 cm^{-1} can be assigned to the symmetric stretching vibration of the aromatic SO_3H [1]. The peak for the asymmetric stretching vibration of the SO_3H is probably located at 1180 cm^{-1}, but it is not clearly distinguishable from other overlapping absorption peaks [2].

2.4. TGA

The thermal stabilities of PES and S-PES were also investigated by thermal gravimetric analysis (TGA) and differential thermal analysis (DTA), as shown in **Error! Reference source not found.**a and b. PES showed only 10% weight loss at around 530 °C, then a sharp weight loss occurred due to the decomposition of the polymer backbone. So, PES is an excellent thermostable polymer. In the case of S-PES, the TGA curve showed a weight loss between 50-100 °C by around 5% due to the evaporation of absorbed water. 5% loss indicates that two water molecules are strongly adsorbed on each sulfonic acid group (λ=2 at normal atmospheric conditions). This weight loss is in good agreement with the endothermic peak from 25 to 100 ˚C on the DTA curve. At around 300 °C, a second weight loss started, possibly owing to the decomposition of the sulfonic acid groups, accompanied by a second endothermic peak near 350 °C on the DTA curve [4]. Finally, the last decomposition stage began around 500 °C, similar to the PES

sample. The wide exothermic peaks from around 100 to 450 ˚C in the DTA curves of both PES and S-PES are probably due to the rearrangement of the polymer chains [5]. S-PES is a promising polymer as a CEM for ED and RED, as they are operated at a temperature lower than 100 °C.

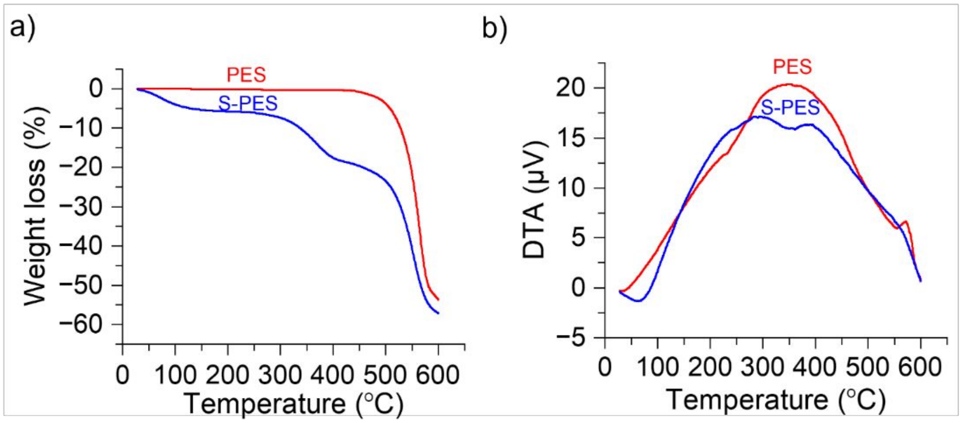

Figure S4. (a) Thermal gravimetric analysis (TGA) and (b) differential thermal gravimetric (DTG) curves for PES and S-PES (*DS* = 36.2%).

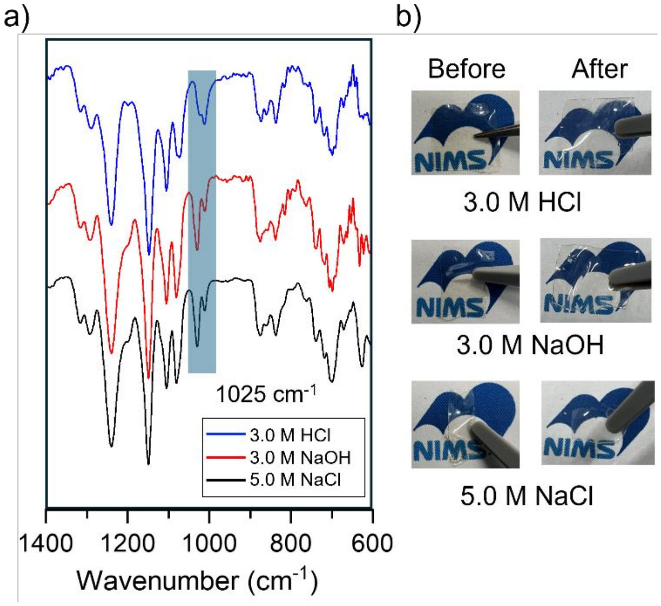

Figure S5. (a) FT-IR spectra for S-PES membrane (8 hours reaction time, *DS* 32.4%) after stirring for 48 hours at 25 °C in 3.0 M HCl, 3.0 M NaOH, and 5.0 M NaCl (b) photo images of the membrane before and after the test.

3. Physical and electrochemical properties

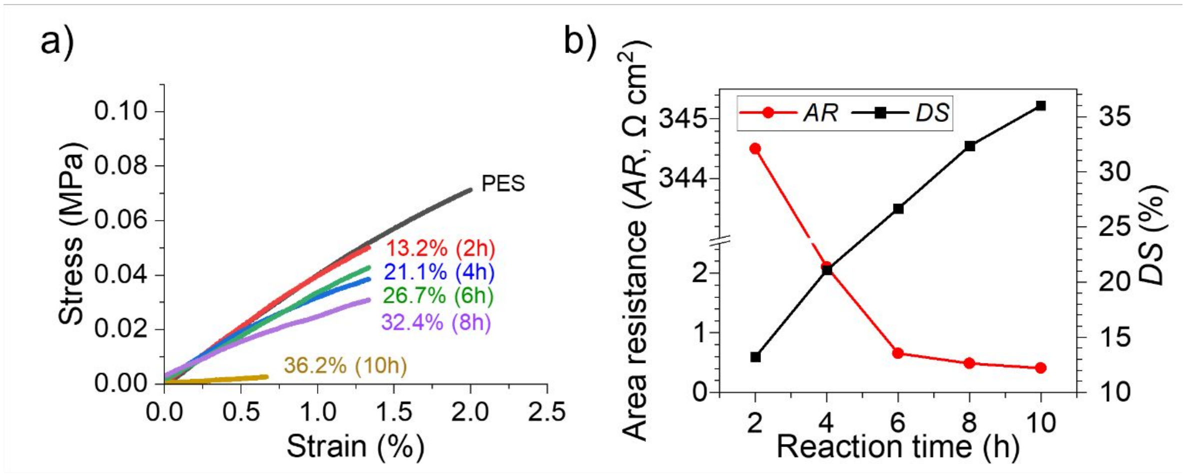

Figure S6. (a) Stress-strain curves for PES and S-PES membranes with different *DS* in the wet state, (b) area resistance and *DS* of the fabricated S-PES membranes as a function of reaction time.

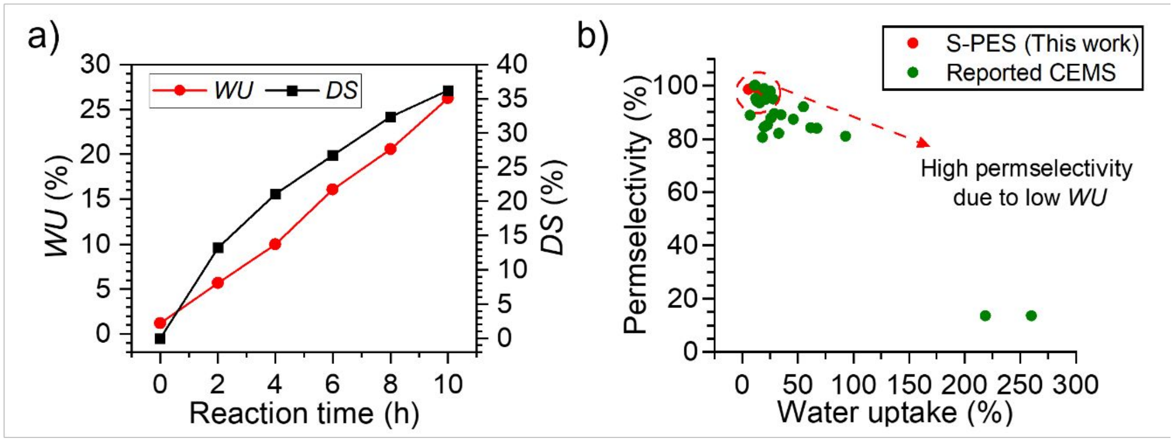

Figure S7. (a) Water uptake and degree of sulfonation of S-PES membranes at different reaction times, (b) permselectivity of S-PES and previously reported CEMs as a function of water uptake.

References

(1) B. C. Johnson, İ. Yilgör, C. Tran, M. Iqbal, J. P. Wightman, D. R. Lloyd, J. E. McGrath. Synthesis and Characterization of Sulfonated Poly(Acrylene Ether Sulfones). *J. Polym. Sci. Polym. Chem. Ed.* **1984**, *22*, 721–737. https://doi.org/10.1002/POL.1984.170220320.

(2) Kim, I. C.; Choi, J. G.; Tak, T. M. Sulfonated Polyethersulfone by Heterogeneous Method and Its Membrane Performances. *J. Appl. Polym. Sci.* **1999**, *74*, 2046–2055. https://doi.org/10.1002/(SICI)1097-4628(19991121)74:8%3C2046::AID-APP20%3E3.0.CO;2-3.

(3) Nolte, R.; Ledjeff, K.; Bauer, M.; Mülhaupt, R. Partially Sulfonated Poly(Arylene Ether Sulfone) - A Versatile Proton Conducting Membrane Material for Modern Energy Conversion Technologies. *J. Memb. Sci.* **1993**, *83*, 211–220. https://doi.org/10.1016/0376-7388(93)85268-2.

(4) Byun, I. S.; Kim, I. C.; Seo, J. W. Pervaporation Behavior of Asymmetric Sulfonated Polysulfones and Sulfonated Poly(Ether Sulfone) Membranes. *J. Appl. Polym. Sci.* **2000**, *76*, 787–798. https://doi.org/10.1002/(SICI)1097-4628(20000509)76:6<787::AID-APP4>3.0.CO;2-1.

(5) Schulz, M.; Seidlitz, A.; Petzold, A.; Thurn-Albrecht, T. The Effect of Intracrystalline Chain Dynamics on Melting and Reorganization during Heating in Semicrystalline Polymers. *Polymer (Guildf).* **2020**, *196*, 122441. https://doi.org/10.1016/j.polymer.2020.122441.
